# Chronic obstructive pulmonary disease, lung function and risk of type 2 diabetes: a systematic review and meta-analysis of cohort studies

**DOI:** 10.1186/s12890-020-1178-y

**Published:** 2020-05-11

**Authors:** Yang Peng, Guo-Chao Zhong, Lingxiao Wang, Lijuan Guan, Ao Wang, Kai Hu, Jing Shen

**Affiliations:** 1 Department of Geriatrics, Fifth People’s Hospital of Chengdu, Chengdu, China; 2 Department of Hepatobiliary Surgery, the Second Affiliated Hospital of Chongqing Medical University, Chongqing, China; 3 Department of Endocrinology, the Second Affiliated Hospital of Chongqing Medical University and Chongqing Clinical Research Center for Geriatrics, Chongqing, China

**Keywords:** Chronic obstructive pulmonary disease, Lung function, Type 2 diabetes, Comorbidity, Meta-analysis

## Abstract

**Background:**

The association between chronic obstructive pulmonary disease (COPD), lung function and risk of type 2 diabetes mellitus (T2DM) remains controversial. We performed a meta-analysis to clarify this issue.

**Methods:**

The PubMed and EMBASE databases were searched. Cohort studies on COPD, lung function and risk of T2DM in adults were included. A random effects model was adopted to calculate the summary risk ratio (RR) and 95% confidence interval (CI). Dose-response analysis was conducted where possible.

**Results:**

A total of 13 eligible cohort studies involving 307,335 incident T2DM cases and 7,683,784 individuals were included. The risk of T2DM was significantly higher in patients with COPD than those without COPD (RR = 1.25, 95% CI 1.16–1.34). Compared to the highest category of percentage forced vital capacity (FVC%), the lowest category of FVC% was associated with a higher risk of T2DM (RR = 1.43, 95% CI 1.33–1.53). Similarly, the summary RR of T2DM for the lowest versus highest category of percentage forced expiratory volume in 1 s (FEV1%) was 1.49 (95% CI 1.39–1.60). Significant linear associations of FVC% and FEV1% with risk of T2DM were found (*P*_non-linearity_ > 0.05); the RR of T2DM was 0.88 (95% CI 0.82–0.95) and 0.87 (95% CI 0.81–0.94) per 10% increase in FVC% and FEV1%, respectively. There was a non-significant relationship between the FEV1/FVC ratio and the risk of T2DM.

**Conclusions:**

Both COPD and impaired lung function, especially restricted ventilation dysfunction, could increase the risk of T2DM. However, these findings should be interpreted with caution due to the limited number of studies, and need to be validated by future studies.

## Background

According to the estimates of the International Diabetes Federation, global diabetes cases were estimated to be 451 million (8.4%) in 2017 and are projected to reach 693 million (9.9%) by 2045 [1]. As the majority of all diabetes cases, the number and percentage of people with type 2 diabetes mellitus (T2DM) are expected to increase. Apart from traditional genetic and environmental risk factors, over recent decades, some novel risk factors of T2DM have received wide attention.

As the fourth leading cause of death worldwide, chronic obstructive pulmonary disease (COPD) plays a prominent part in many chronic conditions and presents in many patients with multi-morbidity [2]. One notable characteristic of COPD is persistent airflow limitation, which is usually measured by spirometry. This test is used to establish the diagnosis and assessment of COPD and is the most widely available test of lung function [3]. Recently, an ample number of longitudinal studies have explored the association between COPD, lung function and risk of T2DM. However, their results have been inconclusive. Most studies found that restrictive ventilation dysfunction as measured by forced vital capacity (FVC) and forced expiratory volume in one second (FEV1), but not obstructive ventilation dysfunction as measured by the FEV1/FVC ratio, was significantly associated with an increased risk of T2DM [4–12]. In addition, some studies have suggested COPD could increase the risk of T2DM [13–17], while other studies showed non-significant results [18, 19].

To our knowledge, a meta-analysis evaluating the abovementioned studies is still absent, and the potential dose-response relationship between lung function and T2DM remains unclear. Therefore, we performed a systematic review and dose-response meta-analysis to evaluate the association between COPD, lung function and risk of T2DM.

## Methods

We reported this systematic review and meta-analysis according to the MOOSE (Meta-analysis of Observational Studies in Epidemiology) guidelines (Supplemental Appendix 1) [20]. Ethics committee approval was not required. There was no review protocol.

### Search strategy and study selection

Two investigators (Y.P. and G.C.Z.) searched the PubMed and EMBASE databases (up to February 2018) using predefined search terms. A detailed description of the search strategy is displayed in the Supplementary Appendix 2. To avoid omitting any eligible study, we performed an updated literature search prior to last submission (March 2020). We also manually searched the references listed in the identified studies and relevant reviews. The language was restricted to English and Chinese. We emailed the original authors for data information when necessary.

Two investigators (W.A. and H.K.) independently reviewed the citations and selected eligible studies based on inclusion and exclusion criteria using Endnote X7 (Clarivate Analytics, PA, USA). We included studies that evaluated the relationship between COPD, lung function and risk of T2DM. The studies identified should contain the incidence of T2DM with adjusted risk estimates and corresponding 95% confidence intervals (CIs). Because both COPD and T2DM are chronic conditions partly caused by aging, and COPD is rarely diagnosed before age 35 years [3]. Moreover, there are different pathogenesis and risk factors from adults in children cases. Therefore, we studied this association in adults and excluded studies limited to children (< 18 years). We also excluded the study of gestational diabetes, type 1 diabetes and other specific types of diabetes.

To depict the cause-effect relationship of COPD and lung function on the risk of T2DM better and to minimize the recall and selection biases, we excluded cross-sectional analyses and case-control studies. Even though a randomized controlled clinical trial (RCT) with high quality could provide more powerful evidence to our conclusion, the searching procedure did not retrieve any RCT on this topic. In consequence, only cohort studies were included in our systematic review. Besides, abstracts, conference proceedings and reviews were also excluded.

Through the initial title and abstract screening, we excluded the articles that were obviously irrelevant to our topic. The secondary screening based on the full-text further excluded the duplicate cohorts and studies without sufficient data. A consensus was reached by consulting a third reviewer (Y.P.) in case of any discrepancy.

### Data extraction and quality assessment

For each eligible study, two independent investigators (W.A. and H.K.) extracted data based on a standard extraction form. The following information was extracted: the name of first author, publication year, study location, sex, follow-up years, sample size, the number of incident T2DM, relevant risk estimates and corresponding 95% CIs, and adjustment factors. The most fully adjusted models were selected to minimize confounding bias. Any disagreement in this process was resolved by discussion with another reviewer (Y.P.).

We assessed the methodological quality of the included studies using the Newcastle-Ottawa Quality Assessment Scale (NOS) [21], with an emphasis on the selection of participants, the comparison of confounders and the evaluation of outcomes. Studies given more than 6 points were deemed to be of high quality in the context of a maximum score of 9 for each study.

### Statistical analysis

In our meta-analysis, hazard ratio (HR), odds ratio (OR) and risk ratio (RR) were regarded as equivalents. A random effects model was employed to calculate the summary RR. Because the study by Lin et al. [13] reported the results of COPD patients with and without exacerbations, we combined the HRs of COPD and COPD exacerbations patients using a fixed effects model. Because the study by Ford et al. [18] only provided COPD data stratified by severity (moderate or severe vs mild), both subgroups were used for comparing the risk of T2DM between patients with and without COPD. Similarly, considering that the studies by Zaigham et al. [4] and Yeh et al. [10] were stratified by sex, we used both sexes in the analysis of the relationship between lung function and incident T2DM. We carried out a fixed effects dose-response meta-regression analysis [22] to explore the dose-response association between both FEV1% and FVC% and the risk of T2DM. A restricted cubic spline function [22, 23] with 3 knots at the 10th, 50th and 90th percentiles was adopted to verify the potential non-linear dose–response nature. A *P*_non-linearity_ was computed by testing the null hypothesis that the estimated value of the second spline was equal to zero.

To quantify the degree of heterogeneity across studies, we selected the Q statistic [24] (significance set at *p* < 0.10) and I^2^ statistic (I^2^ > 75.0%, 50.0–75.0% and < 50.0% signified substantial, moderate and low heterogeneity, respectively). A sensitivity analysis was carried out by omitting one study in turn, and we also performed subgroup analyses by sex and race when appropriate.

Publication bias was assessed by Begg’s and Egger’s tests. The STATA software (version12.0, StataCorp LP, College Station, Texas, USA) was used in all statistical analyses. The statistical significance level was assigned at two-sided *p* < 0.05.

## Results

### Literature search

**Fig. 1:**
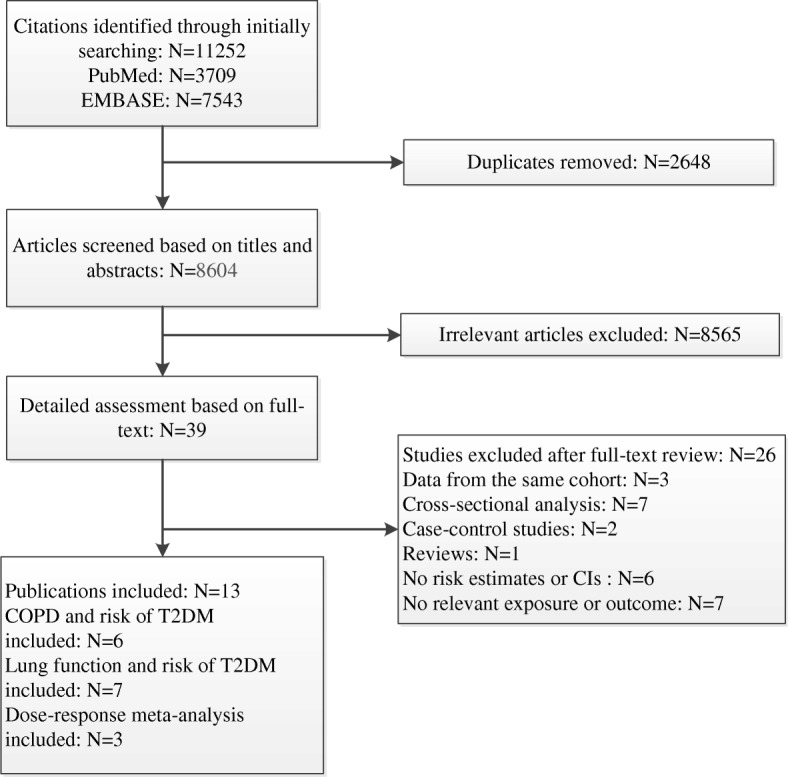
Flowchart of retrieved studies

Of the 11,252 citations identified from literature search, we obtained 8604 articles after removing duplicates. A total of 8565 articles were subsequently excluded after reviewing titles and abstracts. A detailed assessment based on the full text was performed in 39 articles, of which 13 articles were eligible for inclusion. Because of the insufficient data presented in the included studies, only 3 studies were eligible for the dose-response meta-analysis. A detailed description of the screening process and the reasons for exclusion are displayed in the flow chart (Fig. 1).

### Study characteristics

As shown in Table 1, all included articles were prospective or retrospective cohort studies published in the past 15 years, involving 307,335 incident T2DM cases and 7,683,784 individuals. Study locations varied widely from America, Europe (Denmark, Sweden and Britain) to Asia (Japan, Korea and Taiwan). The follow-up durations ranged from 4.7 to 27 years (average follow-up durations: 12.5 ± 8.4 years). Among the studies comparing patients with COPD to those without COPD, most studies included a general population consisting of both males and females, apart from the studies by Song et al. [16] and Rana et al. [17] that only included female health professionals and female registered nurses, respectively. For the studies involving lung function, all the participants were general population and health-screening population. Three studies [7–9] included only males, while the remaining studies included both sexes. In addition, most studies adjusted for identified confounding factors such as age, sex, body mass index, smoking status and comorbidities. The detailed data regarding the results of the included studies is displayed in Table 1.

**Table 1:** The characteristics and outcomes of included studies

Reference	Study location	Follow-up, years	Sex	Sample size/ Incident T2DM	Adjustment factors	Exposure and effect size (95% CI)
Lin et al.,2017 [13]	Taiwan	13	M&F	32,697/1231	age, income, sex, co-morbidities (hypertension, mental disorders, ischemic heart disease, stroke, heart failure, dementia, hyperlipidemia, anemia, Parkinson’s disease, atrial fibrillation, liver cirrhosis, peripheral vascular disease, renal dialysis), anticoagulants, anti-platelet agents, lipid-lowering agents	COPD VS No COPD:1.09 (1.02–1.17) COPDe^a^ VS No COPD: 2.18 (1.88–2.52)
Lee et al.,2013 [14]	Taiwan	5.5	M&F	16,088/884	age, sex, residential area, socioeconomic status, steroid use, hypertriglyceridemia, hypertension, coronary artery disease and cerebrovascular disease	COPD VS No COPD: 1.41 (1.23–1.63)
Sode et al.,2011 [15]	Denmark	27	M&F	7,419,791/292228	age, sex, descent, geographical residency, and level of education	COPD VS No COPD: 1.21 (1.20–1.23)
Song et al.,2010 [16]	U.S	12.2	F	34,056/2016	age, randomized treatment assignment, BMI, physical activity, smoking, alcohol intake, postmenopausal hormone use, family history of diabetes, history of hypertension, history of hypercholesterolemia, total calorie intake, dietary factors	COPD VS No COPD:1.33 (1.09–1.63)
Rana et al.,2004 [17]	U.S	8	F	97,245/2959	age, BMI, sedentary, smoking status, daily alcohol intake, a dietary score variable	COPD VS No COPD: 1.80 (1.10–2.80)
Ford et al.,2004 [18]	U.S	22	M&F	6555/519	age, sex, race, ethnicity, education, smoking status, systolic blood pressure, use of antihypertensive medication, cholesterol concentration, BMI, alcohol use, recreational exercise, non-recreational activity	COPD (moderate or severe) VS No COPD: 1.02 (0.68–1.53); COPD (mild) VS No COPD: 0.66 (0.39–1.12)
Zaigham et al.,2016 [4]	Sweden	26	M&F	27,711/4746	age, height, BMI, smoking status, ESR (log transformed), baseline glucose, cholesterol, physical activity, antihypertensive medication, social class, family history of diabetes, alcohol abuse.	FEV1% in males:≥106.34, 1.0 (reference); 95.57–106.34, 1.06 (0.97–1.17);84.65–95.57, 1.12 (0.95 to 1.33); ≤84.65, 1.48 (1.35–1.63); FEV1% in females:≥106.67, 1.0 (reference); 96.13–106.67, 1.26 (1.04–1.53); 85.14–96.12, 1.26 (1.04–1.53); ≤85.13, 1.45 (1.20–1.75); FVC% in males:≥106.88, 1.0 (reference); 97.42–106.88, 1.06 (0.96–1.17); 87.34–97.42, 1.24 (1.13–1.37); ≤87.34, 1.48 (1.35–1.63); FVC% in females:≥107.50, 1.0 (reference); 97.33–107.49, 1.03 (0.85–1.24); 87.95–97.30, 1.31 (1.09–1.58); ≤87.94, 1.39 (1.16–1.67); FEV1/FVC in males < 70% VS ≥70%:1.06 (0.98–1.16); FEV1/FVC in females < 70% VS ≥70%:0.99 (0.81–1.22)
Oda, E et al.,2016 [5]	Japan	6	M&F	2967/89	age, sex, BMI, current smoking and use of antihypertensive drugs, HbA1c, log hs-CRP	FVC%: 102.00–139.00, 1.0 (reference); 92.00–101.00, 1.05 (0.55–1.99); 54.00–91.00, 1.69 (0.95–3.01); FEV1/FVC: 84.00–99.00, 1.0 (reference); 79 .00–83.00, 1.03 (0.57–1.86);37.00–78.00, 1.67 (0.89–3.12)
Kim et al.,2014 [6]	Korea	4.7	M&F	16,195/640	age, sex, exercise, drinking, smoking habits, hypertension, BMI, waist circumference, fasting glucose level.	FEV1%: 85.80–103.20, 1.0 (reference); 70.70–84.30, 1.12 (0.86–1.47); 65.10–84.10, 1.07 (0.73–1.58); FVC%:84.80–101.20, 1.0 (reference); 75.30–95.10, 1.07 (0.73–1.58); 69.40–79.00, 1.12 (0.86–1.47); FEV1/FVC: 78.00–88.00, 1.0 (reference); 62.00–76.00, 1.12 (0.86–1.47)
Kwon et al.,2012 [7]	Korea	5	M	9220/207	age, BMI, education, smoking, exercise, alcohol, HOMA-IR, cholesterol, triglycerides, and high-density lipoprotein cholesterol	FEV1%: > 119.90, 1.0 (reference); 105.50–119.90,1.08 (0.69–1.70); 94.60–105.50, 1.46 (0.95–2.25);≤94.60,1.58 (1.04–2.39); FVC%:≥109.10, 1.0 (reference); 98.60–109.10, 1.66 (1.05–2.62); 89.40–98.60, 1.11 (0.69–1.80);≤89.40,1.84 (1.19–2.84)
Heianza et al.,2012 [8]	Japan	4	M	5346/214	age, parental history of diabetes, physical activity, smoking status, BMI, hypertension, log-transformed triglycerides, high-density lipoprotein cholesterol, HbA1c	FEV1%:≥104.10, 1.0 (reference); 96.40–104.00, 1.25 (0.79–1.99); 88.80–96.30, 1.76 (1.15–2.69);≤88.70, 1.73 (1.14–2.62); FVC%:≥111.70, 1.0 (reference); 103.90–111.60, 1.03 (0.67–1.58); 95.8–103.8, 1.17 (0.77–1.76);≤95.70,1.39 (0.95–2.05); FEV1/FVC:≥83.10, 1.0 (reference); 80.00–83.00, 1.11 (0.75–1.65); 75.90–79.90, 1.10 (0.74–1.63);≤75.80, 0.98 (0.64–1.50)
Wannamethee et al.,2010 [9]	U.K	20	M	4434/256	age, BMI, smoking, physical activity, alcohol intake, social class, evidence of CHD (undiagnosed),triglycerides, SBP, high-density lipoprotein cholesterol, blood glucose, GGT,CRP, IL-6	FEV1: Highest, 1.0 (reference); Lowest, 1.67 (1.09–2.56); FVC: Highest, 1.0 (reference); Lowest, 1.46 (0.97–2.20); FEV1/FVC:≥82.00, 1.0 (reference); 77.00–82.00, 0.77 (0.55–1.07); 71.00–77.00, 0.87 (0.63–1.21);≤71.00, 0.67 (0.46–0.96)
Yeh et al.,2005 [10]	U.S	9	M&F	11,479/1346	age, race, pack-years of smoking, waist circumference, sport activity index,(FVC% + fasting glucose, HOMA-IR, SBP)	FEV1% in males: Highest, 1.0 (reference); Lowest, 1.70 (1.30–2.10);FEV1% in females: Highest, 1.0 (reference); Lowest, 1.50 (1.10–1.90);FVC% in males: > 108.40, 1.0 (reference); < 90.8, 1.30 (1.00–1.70);FVC% in females: > 114.60, 1.0 (reference); < 95.70, 1.40 (1.10–1.80)

^a^ The study by Lin et al. reported the results of COPD patients with and without exacerbations. *BMI* Body Mass Index, *CHD* coronary heart disease, *CI* confidence interval, *CRP* C-reactive protein, *COPD* chronic obstructive pulmonary disease, *COPDe* COPD patients with exacerbations, *ESR* erythrocyte sedimentation rate, *F* female, *FEV1* forced expiratory volume in 1 s, *FVC* forced vital capacity, *GGT* gamma-glutamyl transpeptidase, *HbA1c* glycosylated hemoglobin, *HDL* high density lipoprotein, *HOMA-IR* homeostasis model assessment of insulin resistance, *IL-6* interleukin-6, *M* male, *SBP* systolic blood pressure, *T2DM* type 2 diabetes mellitus, *U.K* United Kingdom, *U.S* United States

With regard to quality assessment, all included studies were scored six stars or more, implying that the quality of the identified articles was generally good. The detailed scoring process is presented in Supplementary Table 1. In addition, there was no significant evidence of publication bias by Egger’s and Begg’s tests (all *p* > 0.05).

### COPD and type 2 diabetes

Six studies [13–18] were included in the meta-analysis comparing the risk of T2DM between patients with COPD and those without COPD, involving 7,606,432 individuals and 299,837 T2DM cases. Except for the study performed by Ford et al. [18], the other five studies [13–17] all showed a significant association between COPD and the risk of T2DM (Table 1).

**Fig. 2:**
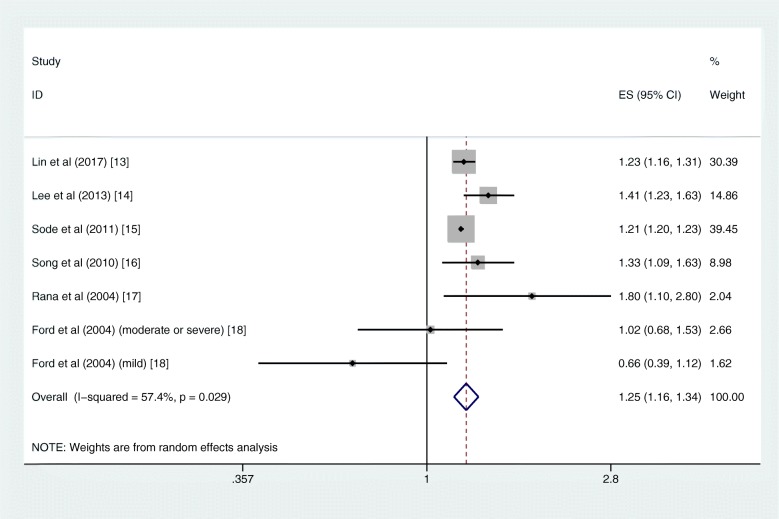
Forest plot showing the RR for T2DM in COPD patients compared with non-COPD population. COPD = chronic obstructive pulmonary disease, RR = risk ratio, T2DM = type 2 diabetes mellitus

The pooled risk of T2DM was higher in patients with COPD than those without COPD (RR = 1.25, 95% CI 1.16–1.34; I^2^ = 57.4%, *P*_heterogeneity_ = 0.029; 6 studies) with moderate heterogeneity (Fig. 2). In the further subgroup analyses divided by sex and race, the increased risks remained consistent. The summary RR for the male subgroup was 1.22 (95% CI 1.18–1.26; I^2^ = 10.5%, *P*_heterogeneity_ = 0.327; 3 studies), and the summary RR for the female subgroup was 1.28 (95% CI 1.21–1.35; I^2^ = 29.8%, *P*_heterogeneity_ = 0.223; 5 studies) (Supplementary Fig. 1). Similar results were found in the subgroup analysis by race; the pooled RRs were 1.30 (95% CI 1.14–1.48) and 1.20 (95% CI 1.01–1.43) for Asian and Caucasian, respectively (Supplementary Fig. 2). The sensitivity analysis did not significantly alter the relationship between COPD and the risk of T2DM.

### Lung function and type 2 diabetes

Seven studies [4–10] were included in the meta-analysis investigating the association between lung function and the risk of T2DM, with 77,352 individuals and 7498 T2DM cases. Overall, due to the most fully adjusted models selected in each included studies, the results derived from these studies are inconsistent (Table 1). Three studies [4, 7, 10] showed significantly higher risk of T2DM in those with restrictive ventilation dysfunction as measured by both FVC% and FEV1%, whereas the other four studies [5, 6, 8, 9] showed non-significant association between FVC% and the risk of T2DM. With regard to the FEV1/FVC ratio and the T2DM risk, only one study [9] showed significant result.

**Fig. 3:**
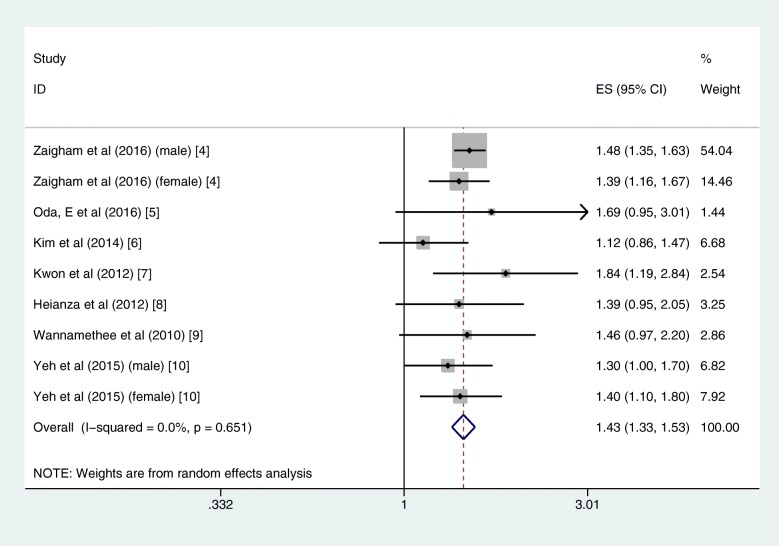
Forest plot showing the RR for T2DM in the lowest versus highest category of FVC%. FVC% = percentage forced vital capacity, RR = risk ratio, T2DM = type 2 diabetes mellitus

**Fig. 4:**
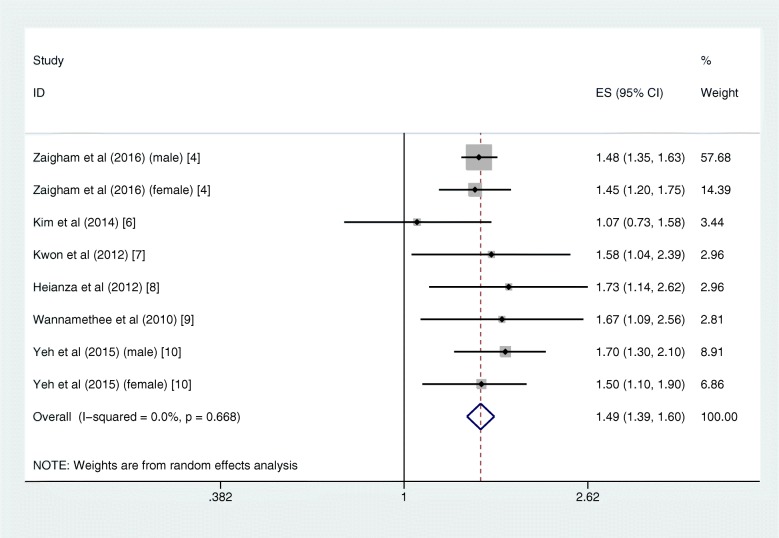
Forest plot showing the RR for T2DM in the lowest versus highest category of FEV1%. FEV1% = percentage forced expiratory volume in 1 s, RR = risk ratio, T2DM = type 2 diabetes mellitus

**Fig. 5:**
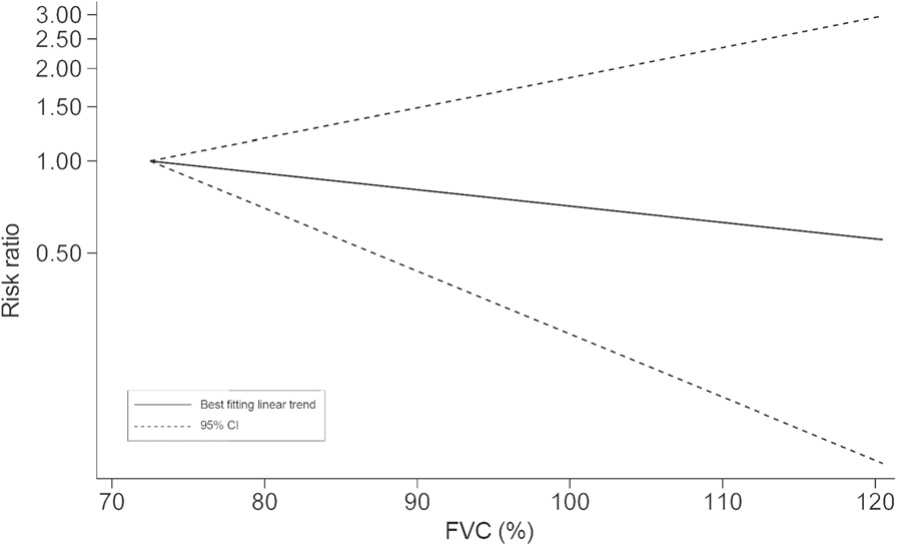
RR for T2DM with increasing FVC% modelled using restricted cubic splines. FVC% = percentage forced vital capacity, RR = risk ratio, T2DM = type 2 diabetes mellitus

**Fig. 6:**
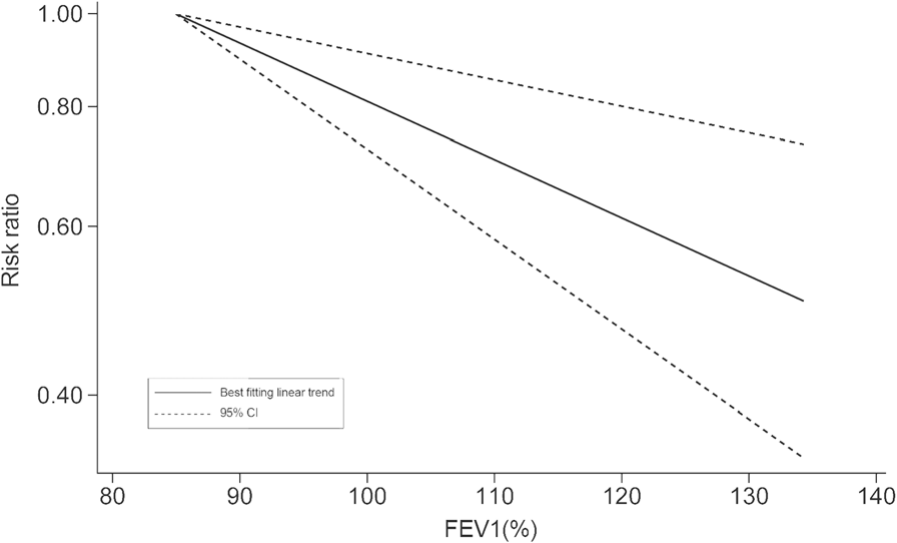
RR for T2DM with increasing FEV1% modelled using restricted cubic splines. FEV1% = percentage forced expiratory volume in 1 s, RR = risk ratio, T2DM = type 2 diabetes mellitus

**Fig. 7:**
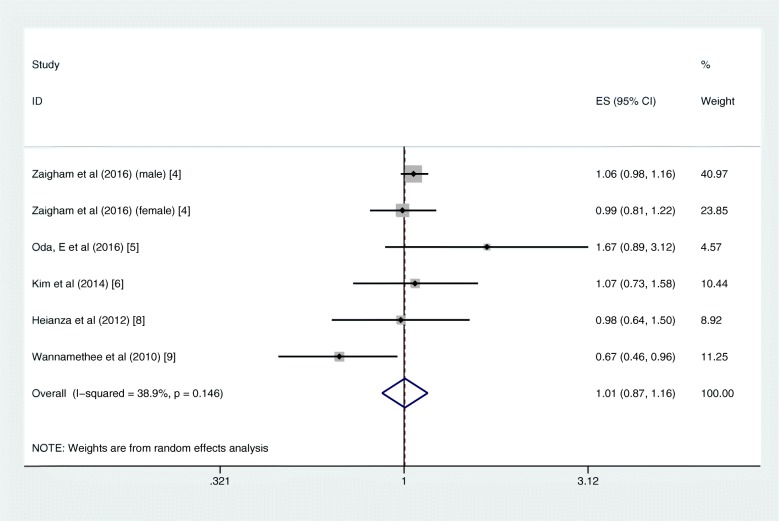
Forest plot showing the RR for T2DM in the lowest versus highest category of FEV1/FVC ratio. FEV1 = forced expiratory volume in 1 s, FVC = forced vital capacity, RR = risk ratio, T2DM = type 2 diabetes mellitus

In quantitative pooled analysis, compared to the highest category of FVC%, the lowest category of FVC% showed higher risk (RR = 1.43, 95% CI 1.33–1.53; I^2^ = 0.0%, *P*_heterogeneity_ = 0.651; 7 studies) with the slightest heterogeneity (Fig. 3). The results for FEV1% were similar, and the summary RR for the lowest versus highest category was almost 1.5-fold (RR = 1.49, 95% CI 1.39–1.60; I^2^ = 0.0%, *P*_heterogeneity_ = 0.668; 6 studies; Fig. 4). The dose-response meta-regression model found significant evidence of linear associations between both FVC% and FEV1% and the risk of T2DM (Figs. 5 and 6). The RR of T2DM for each 10% increase in FVC% was 0.88 (95% CI 0.82–0.95; 3 studies), and the RR for each 10% increase in FEV1% was 0.87 (95% CI 0.81–0.94; 2 studies). Nevertheless, we failed to find a significant relationship between the FEV1/FVC ratio and the T2DM risk (RR = 1.01, 95% CI 0.87–1.16; I^2^ = 38.9%, *P*_heterogeneity_ = 0.146; 5 studies; Fig. 7). The sensitivity analysis did not significantly alter the relationship between lung function and the risk of T2DM.

## Discussion

Our current systematic review and meta-analysis of cohorts regarding the relationship between COPD, lung function and T2DM demonstrated that the incidence of T2DM was higher in COPD patients than in those without COPD. Furthermore, marked inverse associations existed between both FVC% and FEV1% and the risk of T2DM, showing linear associations with each 10% increase in FVC% and FEV1% lowering the risk of T2DM by 12 and 13%, respectively. However, there was no significant link between the FEV1/FVC ratio and T2DM risk, which coincided with previous studies [4–12].

The burden of comorbidities of COPD has been emphasized in recent years, and diabetes is one of the top three comorbidities of COPD related to the highest excess costs [25]. A survey in a German population showed that the prevalence of T2DM was over 28% in COPD patients [26]. Hence, a large number of epidemiological investigations have emerged to explore the possible associations between COPD, lung function and T2DM. The data from the Atherosclerosis Risk in Communities Study and the Cardiovascular Health Study showed that individuals with Global Initiative for Chronic Obstructive Lung Disease (GOLD) stage 3 or 4 COPD and restrictive ventilation dysfunction had a higher prevalence of diabetes (RR 1.5, 95% CI 1.1–1.9; and RR 2.1, 95% CI 1.9–2.5; respectively) [27]. However, most available studies were cross-sectional surveys restricted to a region, which are not generalizable and cannot sufficiently clarify the causal effect.

To our knowledge, this is the first dose-response meta-analysis of cohort studies to quantitatively clarify the association between COPD, lung function and T2DM. A previous meta-analysis by Malte Rasmussen et al. [28] found a higher risk of T2DM in the COPD group (OR 1.17, 95% CI 1.01–1.35); however, this meta-analysis combined four cohort studies and three case-control studies, and subsequent subgroup analyses separating cohort studies and case-control studies did not find any relationship between COPD and T2DM, which was inconsistent with our findings. One possible explanation is that case-control studies could induce an inevitable retrospective bias, and it is not reasonable to combine incidence and prevalence in one meta-analysis.

A noteworthy correlation between COPD and the risk of T2DM has enormously raised interest in studying the underlying biological mechanisms. It is well-known that genetic susceptibility is one of notable characteristics of T2DM, with a 40% life-time risk of T2DM for descendants when a parent has T2DM and the risk increases to 70% when both parents have T2DM [29]. A cohort twin study of 13,649 twins from the Danish Twin Registry suggested that shared genetic factors played a crucial role in comorbidity between COPD and T2DM, and the genetic correlation between two conditions was 43% [30]. Thus, it has been hypothesized that COPD could increase the risk of T2DM by identical genetic factors. However, the specific gene loci are still unknown and need to be thoroughly tested in future studies.

Previous studies have demonstrated that inflammatory markers and oxidative stress could increase the risk of T2DM [31, 32]. Persistent hyperglycaemia promotes the production of reactive oxygen species and activates inflammation mediators, which subsequently increases glucose intolerance [33]. This vicious cycle accelerates insulin resistance and impairment of beta cell function [32–34]. The characteristic pathological change of COPD is a chronic inflammatory response in the respiratory tract and lung parenchyma [3], particularly during acute exacerbations. Moreover, available evidence has found that insulin resistance in COPD patients is associated with inflammation mediators such as C-reactive protein (CRP), interleukin-6 (IL-6) and tumour necrosis factor–α [35, 36]. Based on the above studies, some articles have suggested that enhanced levels of inflammation and oxidative stress were plausible links with insulin resistance in COPD patients [35].

The changes in daily lifestyle, body composition and lipid metabolism due to COPD could also contribute to the development of prediabetes and T2DM. It is obvious that the level of daily physical activity of COPD patients is significantly decreased when compared to healthy control subjects [37, 38]. Individuals with COPD are more inclined to sedentary behaviour and spending less time on activity, especially those with severe cases and elderly patients [37, 38]. Physical inactivity and sedentary behaviour are important risk factors of T2DM [39]; thus, these are probably mediating factors between the two diseases. Owing to less exercise and some drug treatment regimens for COPD, changes in body composition are prevalent. Recently, a multicentre longitudinal observational study found that there was a 3.3-fold higher risk of sarcopenic obesity in patients with COPD than in controls [40]. Obesity, especially abdominal obesity, is a widely accepted risk factor for T2DM [41]. In addition, dysregulated lipid metabolism may mediate the association between COPD and the risk of T2DM [14, 42].

Multiple medication use has been related to the increased risk of new-onset T2DM. Current evidence indicates that inhaled corticosteroids (ICSs) have effects on incident T2DM [43–45]. A longitudinal cohort study in 15,287 newly diagnosed COPD patients demonstrated a higher risk of diabetes in individuals using ICSs (OR 1.23; 95% CI 1.07, 1.47) after adjustment for confounders [45]. According to the latest GOLD guidelines, systemic glucocorticoids is recommended in COPD exacerbations, and ICSs could be used in stable COPD when blood eosinophil counts ≥300 cells/μL [3]. Glucocorticoids could cause decreased beta cell function and insulin resistance [46]. In addition, a predominant circadian cycle of hyperglycaemia in the afternoon and evening has been found in COPD patients prescribed prednisolone [47]. Therefore, we could assume that COPD increases the risk of T2DM, partly due to side effects of corticosteroids.

COPD is characterized by irreversible obstructive ventilation dysfunction, shown as an FEV1/FVC ratio less than 70%. However, in our study, we did not find a significant association between the FEV1/FVC ratio and the risk of T2DM, which implies that the decreased FEV1/FVC ratio is not the cause for the increased new-onset diabetes in COPD. Conversely, our research found that restrictive ventilation dysfunction as measured by FEV1% and FVC% could increase the risk of T2DM in a prominent linear relationship.

It is intriguing not only that T2DM patients have lower lung function levels but also that impaired lung function is an independent predictor of T2DM [11, 12]. Multiple studies have consistently indicated that lower FEV1 and FVC may precede T2DM diagnosis, which may reflect a causal effect of restrictive lung impairment on glucose metabolism [4–7]. Current evidence supports the notion that reduced lung function may contribute to insulin resistance, prediabetes and diabetes [48–50]. Despite the exact pathophysiological mechanisms underlying these relationships remain unclear, some investigators have provided hypotheses that hypoxaemia induced by restrictive lung impairment possibly contributes to glucose intolerance [51]. Moreover, low birthweight might be a common risk factor for reduced lung function and T2DM in adulthood [52, 53].

The strength of our study is that only cohort studies were included in our meta-analysis. Distinct from previous systematic reviews, without the interference of cross-sectional and case-control studies, we could depict the cause-effect relationship of COPD and lung function on the risk of T2DM with the slightest retrospective bias. The NOS scale also showed relatively high quality in all included studies. In addition, as the first dose-response meta-analysis on this topic, we provided data that could quantify the magnitude of the increased risk. Epidemiological studies have usually been restricted by sample size, ethnic diversity and bias, so a single study could be under-powered to reach precise conclusions. In contrast, our study consisted of sufficient samples in a diversity of countries and regions, and thus the results have increased validity and generalizability. Furthermore, the potential confounding factors could be minimized because the most fully adjusted models were selected in each study.

Our study has several limitations. First, we should not neglect the limited number of studies in our meta-analysis. For example, there are only three studies included in the dose-response meta-analysis, which limited our capacity to find potential effect modifiers through subgroup analyses. Second, although we extracted risk estimates from the most fully adjusted models, we still cannot exclude the possibility that our results were subject to residual confounding. Third, most included studies were based on electronic medical record databases, and misclassification is possible due to coding errors and inconsistent diagnostic criteria of COPD and T2DM. Fourth, despite the fact that we found no evidence of publication bias by Begg’s and Egger’s tests, when the number of included studies is less than 10, these tests have insufficient power. Consequently, our results might have been influenced by publication bias. Finally, there was moderate heterogeneity across the studies examining the association between COPD and risk of T2DM, and higher heterogeneity across the studies was observed in different races by subgroup analysis. Therefore, these results should be interpreted with caution.

## Conclusion

In conclusion, our study reveals that patients with COPD or impaired lung function are at an increased risk of developing T2DM; thus, clinical practitioners should pay much attention to the glycaemic level of these patients.

## Supplementary information

**Additional file 1: Supplementary Figure 1.** Forest plot showing the RR for T2DM in COPD patients compared with non-COPD population by sex subgroup. COPD = chronic obstructive pulmonary disease, RR = risk ratio, T2DM = type 2 diabetes mellitus.

**Additional file 2: Supplementary Figure 2.** Forest plot showing the RR for T2DM in COPD patients compared with non-COPD population by race subgroup. COPD = chronic obstructive pulmonary disease, RR = risk ratio, T2DM = type 2 diabetes mellitus.

**Additional file 3: Supplementary Table 1.** The results of quality assessment for included cohort studies.

**Additional file 4.** MOOSE Checklist.

**Additional file 5: Supplementary Appendix 2.** search strategy.

**Additional file 6.** PRISMA 2009 Checklist.

**Additional file 7.** PRISMA 2009 Flow Diagram.

## Data Availability

All data generated or analysed during this study are available from the included studies in this article.

## Abbreviations

CI: Confidence interval
COPD: Chronic obstructive pulmonary disease
CRP: C-reactive protein
GOLD: Global Initiative for Chronic Obstructive Lung Disease
HR: Hazard ratio
ICSs: Inhaled corticosteroids
IL-6: Interleukin-6
MOOSE: Meta-analysis of Observational Studies in Epidemiology
OR: Odds ratio
RCT: Randomized controlled clinical trial
RR: Risk ratio
T2DM: Type 2 diabetes mellitus
FEV1: Forced expiratory volume in 1 s
FEV1%: Percentage forced expiratory volume in 1 s
FVC: Forced vital capacity
FVC%: Percentage forced vital capacity
