# Supplementary material for: Chronic obstructive pulmonary disease, lung function and risk of type 2 diabetes: a systematic review and meta-analysis of cohort studies
Source: BMC Pulm Med. 2020 May 11;20:137. doi: 10.1186/s12890-020-1178-y (PMC7216332; doi:10.1186/s12890-020-1178-y)
Supplement: Supplementary file 3 — Additional file 3: Supplementary Table 1. The results of quality assessment for included cohort studies. [file 12890_2020_1178_MOESM3_ESM.doc]

| **Supplementary Table 1** The results of quality assessment for included cohort studies. | | | | | | | | | | | |
| --- | --- | --- | --- | --- | --- | --- | --- | --- | --- | --- | --- |
| Study | Selection | | | | Comparability | | Outcome | | | | Total Score |
|  | Representativeness of exposed cohort  ☆ | Selection of non-exposed cohort  ☆ | Exposure ascertainment  ☆ | No DM at enrollment  ☆ | Comparable  on confounders  ☆☆ | | Outcome  Assessment  ☆ | Adequate follow-up  (≥5y)  ☆ | | Loss to follow-up rate (≤20%)  ☆ |  |
| Lin et al,2017 | ☆ | ☆ | ☆ | ☆ | ☆ | ☆ | ☆ | | ☆ |  | 8 |
| Lee et al,2013 | ☆ | ☆ | ☆ |  | ☆ | ☆ | ☆ | | ☆ |  | 7 |
| Sode et al,2011 | ☆ | ☆ | ☆ |  | ☆ | ☆ | ☆ | | ☆ |  | 7 |
| Song et al,2010 |  | ☆ |  | ☆ | ☆ | ☆ | ☆ | | ☆ |  | 6 |
| Rana et al,2004 |  | ☆ | ☆ | ☆ | ☆ | ☆ | ☆ | | ☆ |  | 7 |
| Ford et al,2004 | ☆ | ☆ | ☆ | ☆ | ☆ | ☆ | ☆ | | ☆ | ☆ | 9 |
| Zaigham et al,2016 | ☆ | ☆ | ☆ | ☆ | ☆ | ☆ | ☆ | | ☆ |  | 8 |
| Oda, E et al,2016 | ☆ | ☆ | ☆ | ☆ | ☆ | ☆ | ☆ | | ☆ |  | 8 |
| Kim et al,2014 | ☆ | ☆ | ☆ | ☆ | ☆ | ☆ | ☆ | |  |  | 7 |
| Kwon et al,2012 |  | ☆ | ☆ | ☆ | ☆ | ☆ | ☆ | | ☆ |  | 7 |
| Heianza et al,2012 |  | ☆ | ☆ | ☆ | ☆ | ☆ | ☆ | |  |  | 6 |
| Wannamethee et al,2010 |  | ☆ | ☆ | ☆ | ☆ | ☆ | ☆ | | ☆ |  | 7 |
| Yeh et al,2005 | ☆ | ☆ | ☆ | ☆ | ☆ | ☆ | ☆ | | ☆ |  | 8 |
