# Supplementary material for: Chronic obstructive pulmonary disease, lung function and risk of type 2 diabetes: a systematic review and meta-analysis of cohort studies
Source: BMC Pulm Med. 2020 May 11;20:137. doi: 10.1186/s12890-020-1178-y (PMC7216332; doi:10.1186/s12890-020-1178-y)
Supplement: Supplementary file 4 — Additional file 4. MOOSE Checklist. [file 12890_2020_1178_MOESM4_ESM.docx]

**MOOSE Checklist**

From: Stroup DF, Berlin JA, Morton SC, et al (2000) Meta-analysis of observational studies in epidemiology: A proposal for reporting. JAMA 283:2008–2012. doi:10.1001/jama.283.15.2008.

|  | Reported on page | Comments |
| --- | --- | --- |
| **Reporting of background should include** | | |
| Problem definition | 3-4 | The association between COPD, lung function and risk of type 2 diabetes is inconclusive. |
| Hypothesis statement | 3-4 | COPD and impaired lung function could increase the risk of type 2 diabetes. |
| Description of study outcome(s) | 3-4 | COPD and impaired lung function could increase the risk of type 2 diabetes. |
| Type of exposure or intervention used | 4 | COPD and impaired lung function |
| Type of study designs used | 4 | systematic review and dose-response meta-analysis |
| Study population | 4 | Patients with COPD and impaired lung function |
| **Reporting of search strategy should include** | | |
| Qualifications of searchers (e.g. librarians and investigators) | 4 | investigators |
| Search strategy, including time period used in the synthesis and key words | 4 | Supplementary Appendix 2, and the time was up to March 2020. |
| Effort to include all available studies, including contact with authors | 4 | We performed manual search and emailed the original authors when necessary. |
| Databases and registries searched | 4 | PubMed and EMBASE database |
| Search software used, name and version, including special features used (e.g. explosion) | 4 | Endnote X7 (Clarivate Analytics, PA, USA) |
| Use of hand searching (e.g. reference lists of obtained articles) | 4 | We performed manual search. |
| List of citations located and those excluded, including justification | 7 | Figure 1 |
| Method of addressing articles published in languages other than English | 4 | The language was restricted to English and Chinese. |
| Method of handling abstracts and unpublished studies | 5 | Abstracts and unpublished studies were excluded. |
| Description of any contact with authors | 4 | We emailed the original authors for data information when necessary. |
| **Reporting of methods should include** | | |
| Description of relevance or appropriateness of studies assembled for assessing the hypothesis to be tested | None |  |
| Rationale for the selection and coding of data (e.g. sound clinical principles or convenience) | 5 | Multiple investigators independently extracted data based on a standard extraction form. |
| Documentation of how data were classified and coded (e.g. multiple raters, blinding and interrater reliability) | 5 | Multiple investigators independently assessed. |
| Assessment of confounding (e.g. comparability of cases and controls in studies where appropriate) | 5 | The most fully adjusted models were selected to minimize confounding factors. Table 1 |
| Assessment of study quality, including blinding of quality assessors, stratification or regression on possible predictors of study results | 5 | Newcastle-Ottawa Quality Assessment Scale (NOS) was adopted. |
| Assessment of heterogeneity | 6 | Q statistic and I^2^ statistic |
| Description of statistical methods (e.g. complete description of fixed or random effects models, justification of whether the chosen models account for predictors of study results, dose-response models, or cumulative meta-analysis) in sufficient detail to be replicated | 6 | Random effects models and dose-response models were used with detailed description. |
| Provision of appropriate tables and graphics | 7-10 | Figures and Tables. |
| **Reporting of results should include** | | |
| Graphic summarizing individual study estimates and overall estimate | 8-9 | Forest plot |
| Table giving descriptive information for each study included | 7-8 | Table 1  Supplementary Table 1 |
| Results of sensitivity testing (e.g. subgroup analysis) | 9-10 | The sensitivity analysis did not signifcantly alter the relationship. |
| Indication of statistical uncertainty of findings | 8-10 |  |
| **Reporting of discussion should include** | | |
| Quantitative assessment of bias (e.g. publication bias) | 8 | No publication bias by Egger’s and Begg’s tests was found. |
| Justification for exclusion (e.g. exclusion of non-English language citations) | 7 | Figure 1 |
| Assessment of quality of included studies | 8 | All included studies were scored six stars or more, presented in Supplementary Table 2. |
| **Reporting of conclusions should include** | | |
| Consideration of alternative explanations for observed results | 14-15 |  |
| Generalization of the conclusions (i.e. appropriate for the data presented and within the domain of the literature review) | 15 | Patients with chronic obstructive pulmonary disease and impaired lung function are at an increased risk of developing type 2 diabetes. |
| Guidelines for future research | 15 | More studies are needed and clinical practitioners should pay more attention to the glycaemic level. |
| Disclosure of funding source | 16 | Not applicable |
