# Supplementary material for: Chronic obstructive pulmonary disease, lung function and risk of type 2 diabetes: a systematic review and meta-analysis of cohort studies
Source: BMC Pulm Med. 2020 May 11;20:137. doi: 10.1186/s12890-020-1178-y (PMC7216332; doi:10.1186/s12890-020-1178-y)
Supplement: Supplementary file 5 — Additional file 5: Supplementary Appendix 2. search strategy. [file 12890_2020_1178_MOESM5_ESM.doc]

**Supplementary Appendix 2 search strategy**

| **Search strategy for Embase** |
| --- |
| #1 'lung function':ab,ti OR 'pulmonary function':ab,ti OR 'spirometry':ab,ti OR 'fev1':ab,ti OR 'fvc':ab,ti OR 'forced expiratory volume':ab,ti OR 'forced vital capacity':ab,ti OR 'diffusion capacity':ab,ti OR 'chronic obstructive pulmonary disease':ab,ti 189416  #2 'diabetes':ab,ti 778115  #3 'english':la OR 'chinese':la 31491160  #4 #1 AND #2 AND #3 8058  #5 'review':it OR 'letter':it OR 'editorial':it OR 'erratum':it 4518811  #6 #4 NOT #5 7543 |
|  |
| **Search strategy for Pubmed** |
| #1 lung function[Title/Abstract] OR pulmonary function[Title/Abstract] OR spirometry [Title/Abstract] OR FEV1[Title/Abstract] OR FVC[Title/Abstract] OR forced expiratory volume[Title/Abstract] OR forced vital capacity[Title/Abstract] OR diffusion capacity[Title/Abstract] OR chronic obstructive pulmonary disease[Title/Abstract] 121824  #2 diabetes[Title/Abstract] 518859  #3 English[Language] OR Chinese[Language] 26406506  #4 #1 AND #2 AND #3 4235  #5 Review[Publication Type] OR Letter[Publication Type] OR Editorial [Publication Type] OR Published erratum[Publication Type] 4264191  #6 #4 NOT #5  3709 |
