# Supplementary material for: Chronic obstructive pulmonary disease, lung function and risk of type 2 diabetes: a systematic review and meta-analysis of cohort studies
Source: BMC Pulm Med. 2020 May 11;20:137. doi: 10.1186/s12890-020-1178-y (PMC7216332; doi:10.1186/s12890-020-1178-y)
Supplement: Supplementary file 7 — Additional file 7. PRISMA 2009 Flow Diagram. [file 12890_2020_1178_MOESM7_ESM.doc]

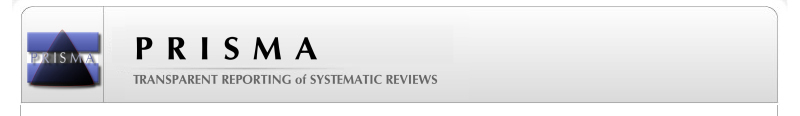
**PRISMA 2009 Flow Diagram**

**Screening**

**Included**

**Eligibility**

**Identification**

Records identified through database searching
(n = 11252)

Additional records identified through other sources
(n = 0)

Records after duplicates removed
(n = 8604)

Records screened
(n = 8604)

Records excluded
(n = 8565)

Full-text articles assessed for eligibility
(n = 39)

Full-text articles excluded, with reasons
(n = 26)

Studies included in **qualitative synthesis**
(n = 13)

Studies included in **quantitative synthesis** **(meta-analysis)**
(n = 13)
